# Development of the Romanian Radar Sensor for Space Surveillance and Tracking Activities †

**DOI:** 10.3390/s22093546

**Published:** 2022-05-06

**Authors:** Liviu Ionescu, Alexandru Rusu-Casandra, Calin Bira, Alexandru Tatomirescu, Ionut Tramandan, Roberto Scagnoli, Dan Istriteanu, Andrei-Edward Popa

**Affiliations:** 1 RARTEL S.A., 011041 Bucharest, Romania; liviu.ionescu@rartel.ro (L.I.); ionut.tramandan@rartel.ro (I.T.); roberto.scagnoli@rartel.ro (R.S.); dan.istriteanu@rartel.ro (D.I.); 2 Faculty of Electronics, Telecommunications and Information Technology, Politehnica University of Bucharest, 060042 Bucharest, Romania; calin.bira@upb.ro (C.B.); alex.tatomirescu@gmail.com (A.T.); andrei_edward.popa@silicon-acuity.com (A.-E.P.)

**Keywords:** space, surveillance, tracking, SSA, SST, LEO, radar, monostatic, debris

## Abstract

The constant increase in the number of space objects and debris orbiting the Earth poses risks to satellites and other spacecraft, both in orbit and during the launching process. Therefore, the monitoring of space hazards to assess risk and prevent collisions has become part of the European Space Policy and requires the establishment of a dedicated Framework for Space Surveillance and Tracking (EU SST) Support. This article presents the CHEIA SST Radar, a new space tracking radar sensor developed and installed in Romania with the purpose of being included in the EU SST sensor network and of contributing to the joint database of space objects orbiting the Earth. The paper describes the processes of design, simulation, and implementation of the hardware and software building blocks that make up the radar system. It emphasizes the particular case of using an already existing system of two large parabolic antennas requiring an innovative retrofitting design to include them as the basis for a new quasi-monostatic radar using LFMCW probing signals. The preliminary design was validated by extensive simulations, and the initial operational testing carried out in December 2021 demonstrated the good performance of the radar in the measuring range and radial speed of LEO space objects.

## 1. Introduction

The new European Union space program for the period of 2021–2027 adopted in April 2021 by the EU Council and European Parliament includes as a key component the Space and Situational Awareness (SSA) program. Under the program, Europe plans to acquire the independent capability to detect, forecast, and estimate risks to people and assets or infrastructure, both on the ground and in space, due to nonfunctional or re-entering artificial space objects; debris resulting from normal launch operations, release events, accidental in-orbit fragmentations, collisions, and explosions; the potential impacts of near-Earth objects; and the effects of space weather phenomena [1].

SSA activities cover three main areas:

- Space Surveillance and Tracking (SST), which is dedicated to detecting, observing, and monitoring man-made Earth-orbiting objects as a tool for collision risk assessment and prevention;
- Space Weather (SWE), which is dedicated to the monitoring and prediction of dangerous levels of radiation and high energetic particles due to solar activity that may negatively impact ground or space-based electronic systems and humans;
- Near-Earth objects (NEO), which is tasked with detecting and monitoring natural space objects passing close to Earth and posing a threat to our planet.

Besides satellites and other spacecraft in good operational state, the outer space around Earth is crowded with space debris. Space debris are composed of satellites that have reached end-of-life, space objects intentionally released as part of missions, rocket parts, frozen satellite propellant, and rocket propulsion systems, as well as fragments that have broken off from space objects due to collisions and explosions [2]. As a result, there is a growing risk of collision for operational satellites and other functional space objects, which may either impair their functionality or result in catastrophic failures.

**Figure 1:**
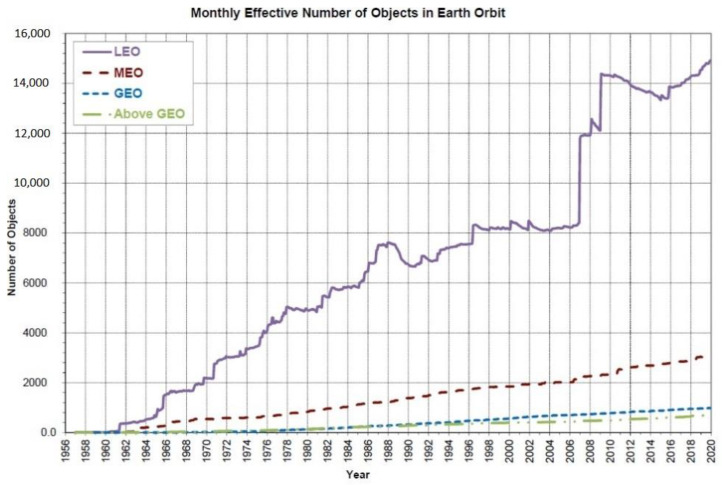
Monthly effective number of catalogued objects in Earth orbit by orbital regime, as catalogued by the US Space Surveillance Network.

According to the US NASA Orbital Debris Program Office, more than 27,000 orbital debris larger than 10 cm are catalogued. The estimated population of particles between 1 and 10 cm in diameter approximates 500,000, while the number of particles larger than 1 mm exceeds 100 million. In 2020, it was estimated that the amount of material orbiting the Earth exceeded 8000 metric tons, with most orbital debris located within the first 2000 km in height above the atmosphere. Extensive studies have concluded that the majority of space debris, operational satellites, and spacecraft are present in the low Earth orbit (LEO), as presented in Figure 1 [3,4].

The European Space Agency (ESA) is currently carrying out its Space Safety Programme (S2P), which aims at mitigating and preventing the impact of hazards from space, protecting activities and infrastructures. Starting in 2021, the closely related SSA element of the Space Programme of the European Union has included complimentary activities carried out within the S2P.

Decision No. 541/2014/EU of the European Parliament and of the Council establishing a Framework for Space Surveillance and Tracking (EU SST) Support states that the specific objectives of the SST support framework are [5]:

(a) assessing and reducing the risks to in-orbit operations of European spacecraft relating to collisions and enabling spacecraft operators to plan and carry out mitigation measures more efficiently;
(b) reducing the risks relating to the launch of European spacecraft;
(c) surveying uncontrolled re-entries of spacecraft or space debris into the Earth’s atmosphere and providing more accurate and efficient early warnings with the aim of reducing the potential risks to the safety of Union citizens and mitigating potential damage to terrestrial infrastructure;
(d) seeking to prevent the proliferation of space debris.

The member countries of France, Germany, Italy, Poland, Portugal, Romania, and Spain, in cooperation with the EU Satellite Centre, are gradually combining their sensor, data processing, and service capabilities to support the operations and decisions of owners and operators of space assets, of civil protection authorities, and of other European entities.

**Figure 2:**
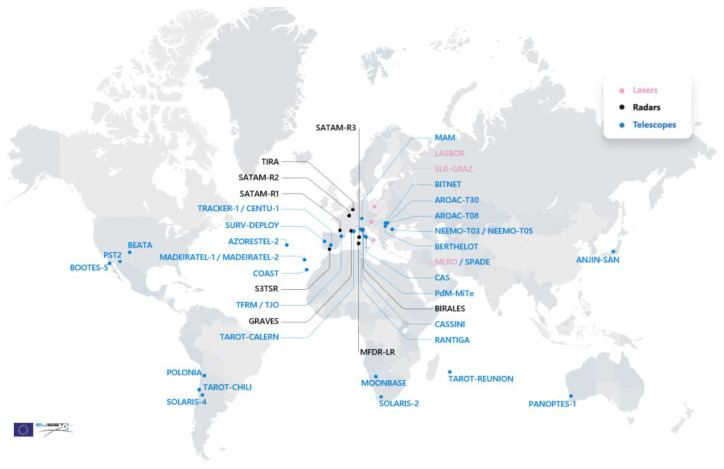
EU SST sensor network status in 2021 Reprinted/adapted with permission from [8] © EU-SST.

The EU SST acquires data from a network of sensors from its member states that includes 38 installations: radars, telescopes, and laser ranging stations (Figure 2) [6]. These sensors survey and track space objects in all orbital regimes: LEO, MEO, and GEO. The acquired data are processed and feed a joint database and are used to maintain a catalogue. The important contribution of the EU SST to space industry applications is illustrated by the fact that more than 210 spacecraft of the European Union, its member states, and European owners and operators are currently protected from the risk of collision in all the orbit regimes. The EU SST also provides more than 100 users with free, added-value services in re-entry and fragmentation analysis [7].

This paper describes the development of the first Romanian Space Surveillance and Tracking radar sensor and is organized as follows: Section 1 presents the European legislative and organizational framework put in place to establish a capacity for monitoring the space environment and the growing number of space objects orbiting the Earth. Section 2 describes the characteristics and performance of some European radar sensors active in space surveillance and tracking. Section 3 provides insight into the lengthy decision-making process to reuse an existing satellite communications installation and to retrofit it to develop a Romanian SST radar. Section 4 presents a general block diagram of the Cheia SST Radar, the assumptions of the design, and its expected performance. Section 5 comprises the results of simulations of radar performance compared to the requirements in the statement of work. Section 6 describes the actual hardware of the implemented radar design and the results of initial tests.

This paper is an extension of a conference paper: Ionescu I., Scagnoli R., Istriteanu D., Turcu V., Cheia Antennas Retrofit to a Space Tracking Radar, European Space Agency 1st NEO and Debris Detection Conference NEOSST1, 2019. Available online at https://conference.sdo.esoc.esa.int/proceedings/neosst1/paper/415/NEOSST1-paper415.pdf (accessed on 24 March 2022). The conference paper presented the conclusions of the first stage of the Cheia Retrofit project (SSA CHEIA PHASE 1 study), containing the general design and simulated performances of the future radar (found in Section 5). This paper brings new content, such as detailed design decisions, the subsequent hardware and software implementation of the radar (Section 4), and the obtained experimental results (Section 6).

## 2. Characteristics and Performance of Some European SST Radars

The feasibility study and design of the intended Romanian SST tracking radar included a survey and comparative analysis of the characteristics and performances of similar installations. The sensor function of the EU SST capability consists of a network of sensors to survey and track space objects in all the orbital regimes (LEO, MEO, HEO, and GEO). The network includes (2021 status) 11 operational radar sensors of the member states of the SST Consortium, three for discovery and surveillance (GRAVES, S3TSR, and BIRALES) and eight radar sensors that also have tracking capabilities (TIRA; SATAM1, 2, and 3; GESTRA; BIRALET; and MFDR-MR and LR) [8].

The characteristics and performances of some radar sensors in the EU SST network are summarized below:

- The GRAVES radar (Grand Reseau Adapte a la Veille Spatiale) is a bistatic radar operating in the VHF band with transmitters located near Dijon and receivers located near Apt, France, operated by the French air force. GRAVES concerns itself with active satellites as a priority, carries out continuous surveys, and is able to acquire simultaneous observation data (angles, Doppler shifts, and Doppler rates) for several objects to an altitude of maximum 1000 km, equivalent to a range of more than 2500 km [9].
- The S3TSR radar (Spanish Space Surveillance and Tracking Surveillance Radar) is a ground-based radar in close monostatic configuration operating at the L band from 1215 to 1400 MHz and capable of providing automatic surveillance and tracking of space objects in LEO from 200 km to 2000 km of orbit height above Earth. The design of S3TSR allows for scalable evolution in size and capabilities by adding multiple *Tx* and *Rx* units. The sensor is fully operational since early 2019 and is providing high-accuracy orbital data of more than 2400 LEO objects included in the catalogue maintained by the Spanish Space Surveillance and Tracking Operation Centre (S3TOC), which also generates autonomous conjunction analysis products [10,11].
- The Italian radar sensor BIRALES (BIstatic RAdar for LEo Survey) is a UHF band (410 MHz) bistatic radar composed of a transmitter antenna situated in Cagliari, Sardinia, and a portion of the Northern Cross Radio Telescope operating as a receiving antenna situated in Medicina, Emilia-Romagna, at a 580 km distance. The transmitter is able to supply a maximum power of 10 kW and uses a 7 m fully steerable wheel-and-track parabolic antenna, with a maximum speed of 3 deg/s. The two installations operate for the space surveillance of debris in the LEO. The radar sensor initially operated in CW mode and performed only Doppler measurements. The system was upgraded to use pulse compression, which enables BIRALES to also perform range measurements [12,13].
- The sensor BIRALET (BIstatic RAdar for LEo Tracking) is a bistatic radar configuration located in Sardinia, Italy. The transmitter is a 7 m fully steerable wheel-and-track parabolic antenna located north of Cagliari that transmits a continuous wave (CW) signal of 10 kW at 410 MHz. The receiving system is the Sardinia Radio Telescope (SRT), a 64 m fully steerable wheel-and-track parabolic antenna devoted mainly to radio astronomical observations located at approximately a 40 km distance from the transmitter operating in the frequency range from 0.3 to 116 GHz. The antenna Gain at 410 MHz is 46.6 dBi, and the efficiency at 410 MHz is 57.7%. During measurement campaigns, the BIRALET has been able to detect objects in orbit with radar cross-section (*RCS*), ranging from 0.13 to 13.4 m^2^ in the range interval between 459 and 1224 km [14,15].

New radar sensors will likely be added to the EU SST network, one of these being the ATLAS SST tracking radar, which has been developed since 2019 in Portugal. ATLAS is a new monostatic radar tracking sensor located at the Pampilhosa da Serra Space Observatory (ErPoB), Portugal. ATLAS uses a 9 m parabolic antenna with 46 dB of gain at 5 GHz and a 5 kW solid-state transmitter. The system operates at 5.56 GHz and aims to provide information on objects in LEO with cross sections above 10 cm^2^ at 1000 km [16].

## 3. Cheia Space Surveillance and Tracking Radar Design Overview

Romania, represented by the Romanian Space Agency (ROSA), is a member of the EU SST consortium and has a significant contribution to the European SST system. ROSA operates the national SST Operational Centre that coordinates the contribution of the Romanian sensors within the consortium. The sensor network currently includes several optical telescopes that have already provided timely and valuable data to the EU SST. ROSA has coordinated and participated in efforts to add a Romanian radar sensor to the consortium’s assets [17].

The choice of adding a tracking radar sensor to the Romanian network for SST took into consideration its ability to operate in almost all weather conditions, day and night, without sensitivity to atmospheric or light pollution. The data output provided by a tracking radar include the target’s position in polar coordinates (range and angles), as well as its radial velocity for a certain segment of its course, improving the information accuracy about its trajectory. As the majority of space debris around Earth is present in the LEO, this domain represents the priority interest for SST data collection activity. For this reason, the design project for the Romanian tracking radar sensor stated the requirement that the range of acquiring and tracking of targets should cover the LEO domain, i.e., 200–2000 km.

The task of developing a Romanian tracking radar sensor for SST applications was carried out by a consortium led by the local company RARTEL, a Romanian–Italian joint venture specialized in satellite services and applications [18]. A preliminary study funded by the ESA (entitled SSA for Romania) presented an inventory of existing facilities and infrastructure with associated technical know-how available at Romanian entities and capable to be integrated in international SSA, especially in SST activities. The survey also pointed to the possibility to develop the first Romanian radar sensor for SST data collection by reusing the parabolic C-band antennas available at the National Satellite Communication Center in Cheia, Prahova County [17].

A subsequent project also financed by the ESA (contract SSA CHEIA PHASE 1) resulted in a study detailing the retrofit of the Cheia antennas (two former satellite telecommunication antennas with diameters of 32 m) to a space surveillance tracking radar system [19]. Phase 1 activities included: a review of the current technical condition of the antennas and of the associated devices and support infrastructure; an analysis of necessary changes and refitting that should be carried out in order to obtain a functional SST radar; and evaluation of options and the decision on the future antenna configuration as a monostatic or quasi-monostatic radar system by using either one or two antennas. The detailed original design of the tracking radar was developed with block diagrams, costs, schedule, and risks up to the level of the preliminary design review. The implementation of the design and the actual development of a working tracking radar for SST is being carried out by RARTEL in the framework of a third contract financed by the ESA as a Cheia Retrofit Phase II activity.

### Radar Site Antennas Opportunities and Constraints

**Figure 3:**
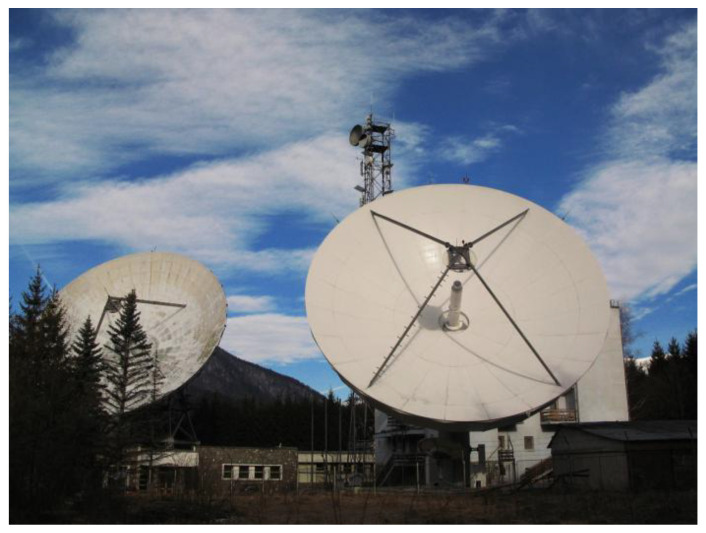
The two antennas at Cheia Satellite Communications Center.

The Cheia Satellite Communication Center in Prahova County, Romania, comprises two decommissioned 32 m diameter parabolic antennas originally installed in 1976 and 1979, respectively, positioned at latitude 45°27′24″ N and longitude 25°56′48″ E at a 900 m altitude (Figure 3). The antennas baseline is 80 m, and each antenna is mounted on top of its own support building.

The most important parameters of the antennas are presented in Table 1 [19]:

One of the main reasons for pursuing this radar development opportunity was the possibility to reuse the two decommissioned antennas and retrofit them to a SST radar. It was considered to be a great advantage because the cost of the antenna system represents an important portion of the total budget of a radar system. Based on the site constraints, the radar possible architectures are presented below [19]:

- C-band quasi-monostatic fadar: In this architecture, both Cheia antennas are retrofitted. One of the Cheia antennas performs transmission on one circular polarization, while the second antenna performs reception on the opposite polarization.

This is a complex solution that eliminates the need for the RF switch in front of the LNA and offers a significant reduction of the power amplifier noise based on the very low coupling between the two antennas. This increases the radar’s sensitivity and, furthermore, allows operation in the CW mode, as well as the pulsed mode. Operation in the CW mode could increase the radar’s sensitivity or allow a reduction in the transmitted power.

- C-band monostatic: In this architecture, only one of the Cheia antennas is retrofitted. It performs both transmission and reception. The radar transmits to the LHCP port and receives at the RHCP port of the same antenna.

The receiving chain must use an RF switch in front of the LNA, and the transmitting chain PA must have a blanking facility so that its final stages are blocked during reception in order to reduce the power-amplifier-induced noise.

The presence of the RF switch, as well as the noise leaked from the transmitter’s PA, strongly reduces the radar’s sensitivity. The radar is only be able to use the pulsed mode.

- C-band bistatic: In this architecture, only one of the Cheia antennas is retrofitted. It performs transmission, while the reception sites must be placed at another site, probably outside Romania. Several reception sites can be used simultaneously.

This architecture is, in principle, the easiest to implement, but it does not represent a truly viable alternative due to its nonindependence. Furthermore, either of the previous two solutions can be used in this configuration if required, for example, in international observation campaigns.

All of the three proposed architectures require an upgrading or a redesigning of the antenna positioning system. To increase the radar’s tracking capabilities, the redesigning of the antenna positioning system was considered of utmost importance.

From the point of view of choosing the modulation of probing signals, the availability of two separate operational antennas allows the implementation of a continuous wave (CW) radar, either frequency-modulated or unmodulated, using both antennas or a pulsed radar that employs just a single antenna. A mixed design of CW and pulsed radars working with the two antennas was also considered as a possibility.

The design of the new radar sensor had to take into account the existing antenna characteristics [19]:

- The two 32 m diameter parabolic Cassegrain beam-waveguide-type antennas were initially designed for large bandwidth operation in the range 3.6 to 6.4 GHz. The antenna main reflector, subreflector, and feed waveguide were improved to achieve the largest possible gain and the best parameters in this bandwidth. The antenna analysis determined that any modification of the antenna system would incur high costs and likely lower the antenna merit factor (G/T ratio); the conclusion was to employ them without any customization.

Within the C band, the International Telecommunication Union (ITU) allocates for Radiolocation services in Region 1 the sub-band of 5250–5850 GHz (ITU Radio Regulations, Edition 2012). Investigations were performed on the composite feed-antenna assembly in this sub-band, and the results showed a very good voltage standing wave ratio (VSWR) of 1.02 at 5840 GHz. Based on the antenna and antenna feeder system measurements, the optimal 20 MHz bandwidth was determined as 5830–5850 GHz; therefore, this bandwidth was selected for the operation of the new Cheia tracking radar.

- The originally designed transmitted-power-handling capability of the antennas in the 6 GHz band using the original structure of the feed was limited to 10 kW CW. Radar modelling showed that 2.5 kW of CW-transmitted power provided satisfactory dimensions of the discovered space objects. In the CW mode of operation, the radar would be able to detect and track objects with dimensions of over 40 cm in LEO at a maximum of 2000 km altitude. In the pulsed mode, using a duty cycle of 30% would increase the dimensions of discovered objects by a factor of three.
- The original antenna positioning system provided a positioning and slewing velocities limited to 0.3°/s on both axes, which was considered to be insufficient for tracking fast space objects. It was determined that the technically obsolete existing system should be reformed and upgraded by replacing main parts of the driving mechanism in order to increase the tracking and slewing speeds to 1.0°/s on each axis.
- An analysis of the results to be achieved in terms of separation between objects close to each other in orbit showed that, as the antenna beamwidth at the selected operating frequency was 0.11°, the angular resolution was limited to 0.06°. Even if the radar system would not use monopulse technology to improve the angular accuracy, taking into account the limited antenna positioning and slewing speed, the radar would provide good results in tracking LEO objects based on TLE data.
- The tracked radar targets must be located within a limited visibility domain to be detected. Each antenna has an azimuth scanning range of ±170° around south and 0° to 92° in elevation. Additional limitations that are due to existing buildings, installations, trees, or other natural terrain features in the close vicinity of the antennas were determined during in situ tests.

## 4. Tracking Radar Design

### 4.1. Design Assumptions

The original design of the new SST radar was carried out based on the following hypotheses:

- Operation with two antennas in quasi-monostatic architecture with an 80 m baseline: In this setup, the Cheia 1 antenna was used for transmitting (*Tx*), and the Cheia 2 antenna was used for receiving (*Rx*).
- While the antenna bandwidth allowed functioning in both radar S and C bands, the frequency range chosen for radar probing signals was 5830–5850 MHz because, in the C band, antenna performances are higher, and this interval was also very close and partly coinciding with the antenna specified bandwidth of 5845–6425 MHz.
- Dual mode modulation capability: The basic operation scenario was continuous wave (CW) modulation, which offers superior performances. The frequency-hopping pulsed (FH-P) mode was also investigated, which offers theoretically superior performances in a dense target situation and mitigates the risk of possible interference between the *Tx* and *Rx* antennas.
- Transmitted power: For the output power of the *Tx* amplifiers, the value of 2.5 kW was finally chosen (−3 dB from the 5 kW saturation point of the PA). This was an optimum value relative to the budget and the minimum size of detectable objects.
- Improved velocity of the antenna positioning system: The retrofitted radar antennas would be capable of superior performances compared to the originals by installing new drive motors and a tracking system, resulting in an increase of the slew velocity above the original 0.3°/s to the target of 1°/s.

### 4.2. Estimated Theoretical Performance of The Considered Solution

The system was designed to track all LEO-resident objects (200–2000 km) within the tracking capabilities of the antenna and having sizes above the following perigee-altitude (*h*_*p*_)-dependent diameter envelope (*d*_*min*_), with *h*_*ref*_ = 1400 km and *d*_*ref*_ = 10 cm [20]:

(1) $$d_{min}=max\left\{\sqrt{\frac{h_{p}^{4}}{h_{ref}^{4}}d_{ref}^{2}},\;1\mathrm{cm}\right\}$$

**Figure 4:**
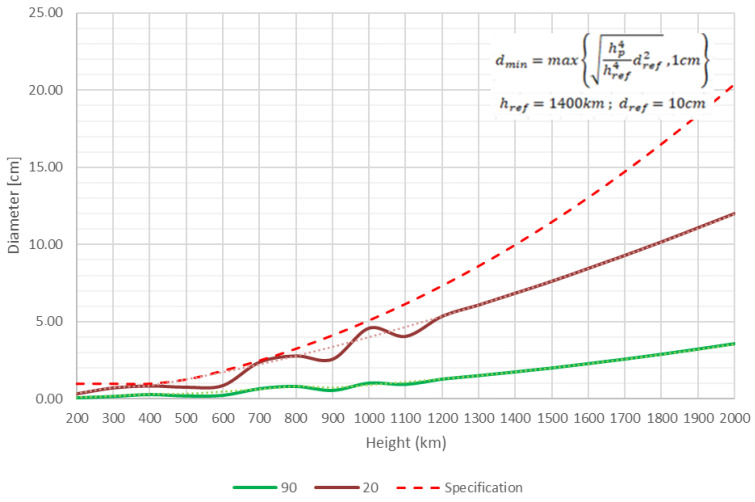
Estimation of minimum size of the target detected in CW mode at two elevation angles (90 and 20 deg.) compared to requirements.

The simulated tracking capabilities of the radar compared to the ESA requirements, are presented in Figure 4.

The capabilities were derived based on the radar equation [21]:

(2) $$RCS_{min}=\frac{{\left(4\pi \right)}^{3}\cdot k_{B}\cdot T_{Rx\;Ant}\cdot B_{Rx}\cdot F_{Rx}\cdot L_{Rx}\cdot SNR_{min}}{P_{Tx}\cdot G_{Tx}\cdot G_{Rx}\cdot \lambda^{2}}\cdot R_{Rx}^{2}\cdot R_{Tx}^{2}$$

and assuming a parabolic antenna gain:

(3) $$G_{Tx}=G_{Rx}=0.6\cdot {\left(\frac{\pi D}{\lambda }\right)}^{2}\approx 63\;\mathrm{dBi}$$

a parabolic antenna noise temperature:

(4) $$T_{Rx\;Ant}=60\;K$$

a spherical reference target of diameter *D*_*sph*_ with a radar cross-section (*RCS*):

(5) $$RCS_{sph}=\pi \cdot \frac{{\left(D_{sph}\right)}^{2}}{4}\left(\mathrm{in}\;\mathrm{the}\;\mathrm{optical}\;\mathrm{region}\frac{\pi D_{sph}}{\lambda }>5\right)$$

an average transmitted power:

(6) $$P_{Tx}=64\;\mathrm{dBm}=2.5\;\mathrm{kW}$$

and a receiver capable of delivering a signal-to-noise ratio (*SNR*) of:

(7) $$SNR_{min}=13.2\;\mathrm{dB}\left(P_{d}=0.9\;;P_{Fa}=10^{-6}\right)$$

for an input signal level of:

(8) $$P_{Rx\;min}=-148\;\mathrm{dBm}$$

To achieve these drastic requirements, a low additive phase noise solid-state power amplifier (SSPA) transmitting LFM (linear frequency modulation) CW, was used in conjunction with a receiver having a bandwidth of:

(9) $$B_{Rx}=20\;\mathrm{Hz}$$

This very narrow receiver bandwidth was implemented through a large fast Fourier transform (FFT). A pulsed solution was considered as well, but the challenges of pulsing a high-power SSPA proved impossible to meet reliability criteria in the present technological state.

### 4.3. Radar Block Diagram and Operation

**Figure 5:**
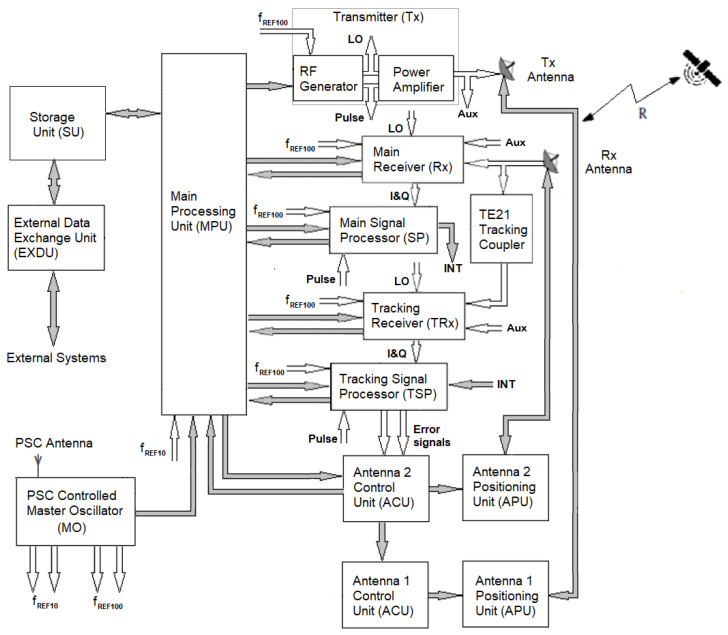
General diagram of Cheia SST radar.

Figure 5 illustrates the general block diagram of the radar. Data flows (digital signals) are presented as grey arrows, while the analogue signals are presented as white arrows. The radar used a flexible architecture that was able to operate both in CW mode by transmitting LFMCW probing signals and in pulsed mode by transmitting long frequency-hopping chirp pulses (FH-P). Due to the actual technological limitations of the solid-state power amplifier, in the present implementation only LFMCW probing signals were transmitted. The following paragraphs describe the main radar subsystems in terms of the blocks presented in Figure 5. The blocks from Figure 5 are referred to in bold letters.

#### 4.3.1. Transmitter

A transmitter (*Tx*) generates the radar probing signals. Its subsytems are an RF generator, a power amplifier, and a master oscillator (PSC MO).

A low-noise RF generator produces a CW triangular LFM signal and a trigger pulse (pulse), indicating the start of each slope of the triangular FM modulating signal. The parameters of the LFM signal are set by a main processing unit (MPU), which configures the RF generator through SCPI commands. To achieve a very high frequency stability, the RF generator is externally driven by the very low-noise, high-stability reference signal (f_REF100_) generated by a positioning-satellite-constellation (PSC)-controlled master oscillator (MO).

The RF generator’s CW LFM output signal is amplified to the required power level by a linear solid-state power amplifier (SSPA) and then applied to the LHCP port of a 6 GHz orthomode transducer (OMT) and radiated as left-hand circular polarization waves through the *Tx* antenna. To achieve a high linearity and a low additive phase noise, the SSPA is used at −3 dB from saturation power.

A part of the RF generator output signal is extracted in order to be used as a local oscillator (LO) signal in both receivers (main receiver and tracking receiver). The LO is extracted through a divider.

The pulse trigger pulse starts ADC acquisition in both the main signal processor (SP) and the tracking signal processor (TSP), belonging to the receiver.

#### 4.3.2. Receiver

The receiver subsystem amplifies, demodulates, filters, and digitally converts the *Rx*-antenna-received signals, preparing them for digital processing. The radar uses two receiving chains, one for the sum channel, or the main receiver (*Rx*), and one for the delta channel, or the tracking receiver (TRx). Each receiver comprises an analog front end and a digital back end. The tracking receiver (TRx) is used for autotracking and for processing the error signals produced by a TE21 tracking coupler installed in the *Rx* antenna feeder.

The sum channel echo signals are received at the RHCP port of a 6 GHz OMT of the *Rx* Antenna and then are fed to the input of the main receiver. They are filtered, frequency translated and demodulated using the LO signal, and amplified to the value required by the optimal operation of an analog-to-digital converter (ADC). To mitigate the increase of phase noise and interferences due to *Tx*–*Rx* coupling through antenna spill-over, obtrusive objects, or transmission mismatch, the main receiver (*Rx*) employs digital self-interference mitigation phase noise cancellation techniques.

The delta channel error signals are received at the RHCP port of a TE21 tracking coupler installed in the *Rx* antenna feeder. They are processed in the same way as the echo signals.

The main receiver (*Rx*) digital baseband output signal is fed to the main signal processor (SP), while the tracking receiver (TRx) digital baseband output signal is fed to the tracking signal processor (TSP) for subsequent processing.

#### 4.3.3. Signal Processor

A signal processor detects the target’s presence and extracts its parameters. The radar uses two processing chains, one for the sum channel, or the main signal processor (SP), and one for the delta channel, or the tracking signal processor (TSP).

The main signal processor (SP):

- Time stamps the input signal;
- Translates the input signal into the frequency domain by using a large short-time Fourier transform;
- Detects the presence of the target through a continuous false alarm rate (CFAR) algorithm;
- Processes the detected target’s frequency domain data in order to extract the target’s range, doppler, and signal-to-noise ratio (*SNR*).

The main signal processor (SP) target’s data are sent to the main processing unit (MPU) for storage, TDM formatting, and display.

The intermediary extracted data INT (detected target’s frequency, its associated complex value, and the corresponding *SNR*) are sent to the tracking signal processor (TSP) to assist in the processing of the delta channel signal. The INT data are sent to the TSP every half-modulation period.

The tracking signal processor (TSP):

- Time stamps the input signal;
- Translates the input signal into the frequency domain by using a large short-time Fourier transform;
- Processes the delta channel frequency domain data in order to extract the complex delta channel signal corresponding to the target;
- Uses the INT data to detect if a tracking lock condition is present and to normalize the azimuth and elevation antenna-positioning error data;
- Converts the azimuth and elevation antenna-positioning error data into analog signals, as required by the antenna 2 control unit (ACU).

#### 4.3.4. Radar Management Unit

The management of radar activity is performed by the main processing unit (MPU). This is a processing system that runs management and control software. Its functions are divided into several classes.

- The campaign preparation functions handle the tracking requests by computing the most probable trajectory of the object based on catalogue data, by determining the visibility windows of the specified objects, and by scheduling the observations.
- The operation functions set the initial data and operational parameters for each radar subsystem. Additionally, they display the tracked object’s estimated position (based on catalogue data) and the tracking errors at the end of the tracking process.
- The control functions check the full system status and perform maintenance tasks.

The management and control software running on the MPU has a software architecture based on microservices. Each microservice has a specialized behaviour and is able to work independently. Each piece of equipment is paired with a corresponding equipment microservice and is integrated into the system through a configuration database.

The targets’ measured data is stored into a storage unit (SU).

The measured data for the selected targets are sent at the operator’s request to external entities over virtually private networks (VPNs) through an external data exchange unit. An outside entity can be represented by a supplemental computation center, an encrypted data center, or another infrastructure belonging to an ESA member state. Protected access to the output data of the radar is administered by the external data exchange unit by employing secured user accounts.

#### 4.3.5. Antenna Control Unit

The azimuth and elevation movement of the *Tx* antenna and *Rx* antenna are controlled by two antenna positioning systems: antenna positioning system 1 (APS1), comprising an antenna 1 control unit (ACU) and an antenna 1 positioning unit (APU), and antenna positioning system 2 (APS2), comprising an antenna 2 control unit (ACU) and an antenna 2 positioning unit (APU). APS1 and APS2 are synchronized by the main processing unit (MPU).

During auto-track, the error signals control the antenna 2 control unit (ACU), which in turn controls the movement of the *Tx* Antenna through the antenna 1 control unit (ACU) in order to maintain positioning synchronization of the two antennas.

## 5. Predicted Performances

### 5.1. Quantity and Quality of Detectable Targets

Before the radar design was implemented, extensive simulations of its performance compared to the requirements in the statement of work were carried out. As expected, the radar was capable of detecting and tracking objects that fly through the visibility field of the *Tx* antenna. The quantity of these targets was predicted using the ESA’s “PROOF-2009” software as a simple simulation environment. Spreadsheet processors were used to additionally post-process and plot the obtained data. The signal characteristics of the planned CW radar were allocated to the settings of the corresponding low duty factor pulsed radar in terms of detection capabilities because PROOF-2009 is intended for the latter type of radar [22]. The simulation mode was set to “Statistical”. The precise parameters of the Cheia topographical coordinates and the mandatory radar characteristics were introduced [23,24]. The simulations had a start time of 09:00 UTC, 30 March 2018, and the duration was fixed to 24 h.

**Figure 6:**
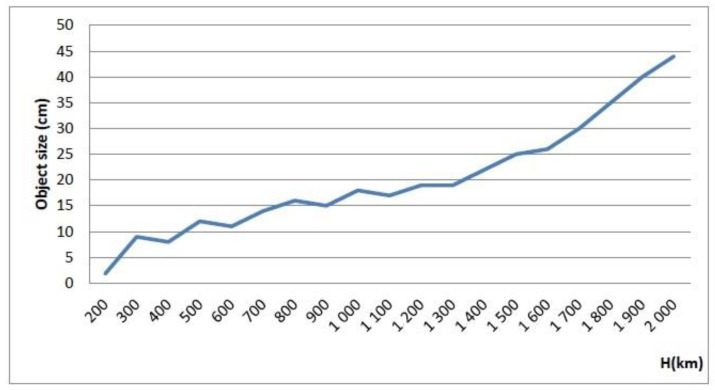
Minimum size of detectable targets according to the orbital altitude in CW mode [19].

**Figure 7:**
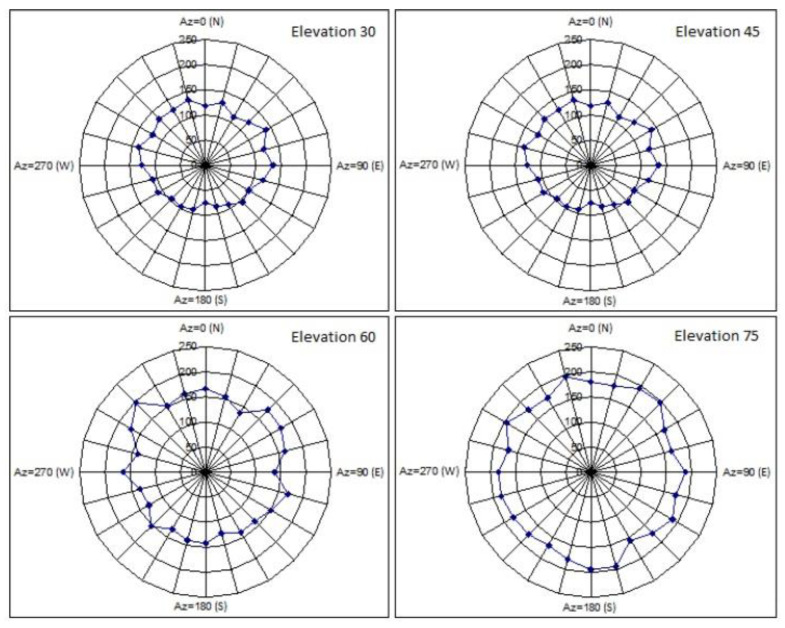
Estimated number of targets trackable in CW mode at varying elevation angles [19].

Figure 6 illustrates the lowest detectable target size relative to the orbital altitude in the CW operation. Figure 7 illustrates the outcomes of the PROOF-2009 modelling, presenting the number of detectable targets for 24 h at varying altitudes in the CW operation.

### 5.2. Mean Duration of the Observed Track

The simulation comprised a 24 h interval starting at 30 March 2018 09:00 UTC and took into consideration space targets passing above the lowest clear elevation angle of the radar, which was 20°, in ranges between 200 km and 5900 km at the Cheia location.

Regarding the number of passing objects possible to be tracked with actual antenna speed parameters, the results were the following [19]:

- The total daily number of trackable targets was 47,068, which signified more than 98.16% of the number of passing space objects.
- For orbital altitudes between 750 and 900 km, the number of passes was 14,836, which was equivalent to 30.94% of the total number of passing space targets. From this number, 4394 were detectable distinct satellites, which represented 32% of the total number of detectable satellites.
- For orbital altitudes above 2000 km, the number of passes was 1747, which was approximately 3.65% of the total number of passing space targets.
- The highest density of trackable objects occurred at three adjacent orbital altitudes: 750 to 800 km, 800 to 850 km, and 850 to 900 km altitudes above Earth.

Other values of interest were the number of space objects that had several passes in the observed sky sector for 24 h and the average duration of one pass. To this purpose, the mean, the standard deviation, and the median for angular arc tracking and track-time were computed:

- For orbital altitudes from 750 to 800 km, 1471 objects had more than one passing, and only three satellites have one passing. The average and median times per passing were 1.95 min and 1.86 min.
- For orbital altitudes from 800 to 850 km, 1561 objects had more than one passing, and only one satellite had one passing. The total number of objects with two or more passings in the 800 to 850 km orbital regime was 1561, and only one satellite had one passing. The average and median times per passing were 2.25 min and 2.08 min.
- For orbital altitudes from 850 to 900 km, 1355 objects had more than one passing, and only three satellites had one passing. The average and median times per passing were 2.59 min and 2.32 min.

The estimations shows that the minimum mean tracking period of a space object that could be repeatedly discovered by the radar was at least 1.86 min with an average delay between tracks of at least 360 min for the same object.

The average antenna repositioning time from the end of a track to the expected start position of the next scheduled track was 4 to 6 min at the actual moving speed of the antenna.

The total average duration required to track one space object was a 6 to 8 min value resulting from the tracking duration of the present target added to the duration for calculating if the next target could be tracked, as well as repositioning time to the start of the following track. For orbital altitudes between 750 and 900 km, the estimated number of daily trackable targets was 180 to 240, out of which the largest number of distinct targets tracked in two passes was between 90 and 120. Should the antenna positioning speed be doubled, the number of tracked targets could be increased by 25% to 40%.

## 6. Initial Tests and Results

The initial tests of the new Cheia SST Radar were carried out during the month of December 2021. The radar subsystems assigned to the tracking function of the antennas were not yet functional. The antennas were oriented in azimuth and elevation by the antenna positioning units according to the projected calculated trajectories of the targeted spatial objects.

The hardware configuration used by the radar sensor was as follows:

- The RF generator was implemented using an SMA-100B Rhode-Schwarz low-noise Microwave Generator, f = 5830–5850 MHz with a phase noise of −125 dBc/Hz and triangular linear frequency modulation that was externally synchronized by a customized GNSS-Controlled Master Oscillator manufactured by Quartzlock (United Kingdom) with an Allen frequency deviation of 5·10^−12^ (at 10 s) and a phase noise of −170 dBc/Hz at 10 KHz offset.
- The power amplifier had a saturation power of 5 kW. It was a custom-made low-noise SSPA manufactured by TTI Norte (Spain) [25]. This high-power SSPA was not used during the initial tests, as it was not available at that time, so the original 150 W power amplifier of the transmitting antenna was used.
- The receiver was a custom-made receiver produced by Ad-Hoc Telecom Solutions (Romania), while the signal processor was a computer featuring four high-performance 16 bit ADCs and a Tesla V100 Graphic Processing Unit (GPU); both the hardware and software were customized by Silicon Acuity (Romania) [26].
- The antenna positioning system was designed and manufactured by Antech Space (Italy) [27].

The parameters of the initial radar tests were:

- Output power: 51 dBm (125 W). It would be increased to 67 dBm (5 kW) after installing the new SSPA;
- Frequency band: 5830–5850 MHz;
- Operating mode: CW (continuous wave);
- Modulation: triangular LFM (triangular linear frequency modulation);
- Antenna gain: 63 dBi;
- Antenna count: two (one for *Tx* and one for *Rx*);
- Receiver noise factor (F_*Rx*_): 1.1 dB;
- Processing loss (L_*Rx*_): 40 dB. This high value was due to the high level of the phase noise of the PA. This increased the noise threshold of the receiver through antenna coupling;
- Antenna noise temperature (T_ant_): 56 K;
- Detection signal-to-noise ratio (*SNR*): 6 dB (min).

A number of five dead spatial objects were targeted, pending the operator’s approval, to emit 5.85 GHz probing signals towards other active objects:

- COSMOS 2392 obsolete satellite (NORAD id 27470, International Designator 02037A) [28].
- ENVISAT obsolete satellite (NORAD id 27386, International Designator 02009A) [29].
- RADARSAT payload (NORAD id 23710, International Designator 95059A) [30].
- SS-18 R/B rocket body (NORAD id 25695, International Designator 99021C) [31].
- DELTA 1 R/B rocket body (NORAD id 13778, International Designator 83004B) [32].

The two-line elements (TLE) input data were generated by the management and control software within the MPU system by using international space objects catalogues.

In Table 2 are presented the measured ranges and radial speeds derived from the received Doppler frequencies for the five objects in LEO targeted during the initial tests of the Cheia radar.

**Figure 8:**
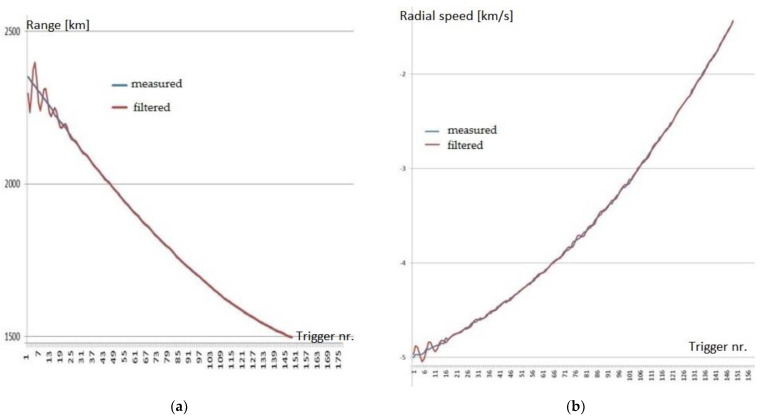
(**a**) Measured and filtered range values for COSMOS2932; (**b**) speed derived from measured and filtered Doppler values for COSMOS2932.

The values resulting from the measurements and after applying an alpha–beta filtering process for range and Doppler speed were in good accordance with the estimated ones (calculated from the two-line elements data) [33]. In Figure 8a,b the two sets of values for range and Doppler speed are plotted for spatial object COSMOS2932. On the x-axis, the time unit is the trigger timing of the analog-to-digital converter, with the full scale of 151 triggers corresponding to approximately 237 s of measurement time.

**Figure 9:**
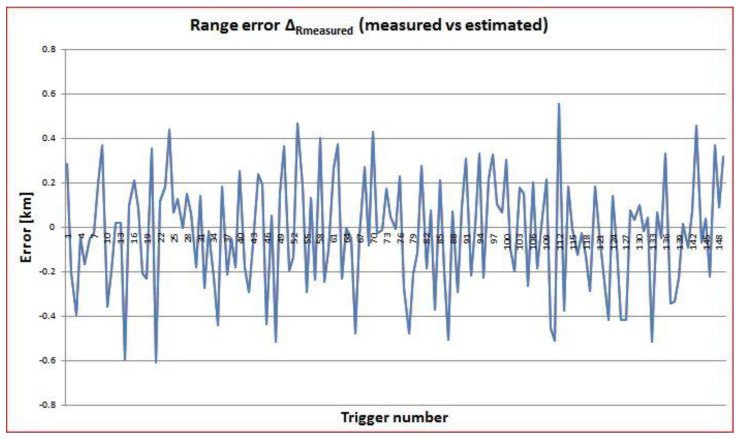
Difference between the measured and the TLE-computed values for range as a function of trigger number (time) for COSMOS2932.

**Figure 10:**
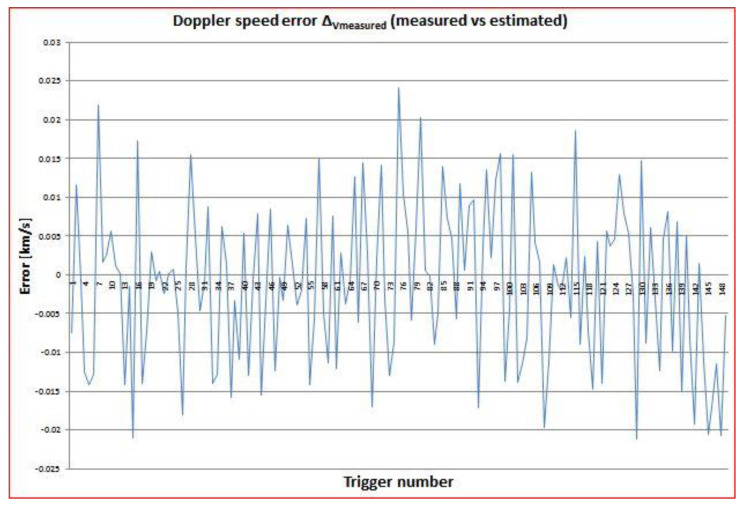
Difference between the measured and the TLE-computed values for Doppler speed as a function of trigger number (time) for COSMOS2932.

In order to estimate the accuracy of the measured values for range and Doppler speed in comparison with the theoretical estimated values, the differences Δ_Rmeasured_ = R_measured_ − R_estimated_ and Δ_Vmeasured_ = V_measured_ − V_estimated_ were also computed and plotted in Figure 9 and Figure 10. The estimated values were obtained from the TLE values. It was shown that these errors were quite small. The range error was inside an interval of less than ±0.6 km compared to the mean range of 1923 km for COSMOS2932. The Doppler speed error belonged to an interval of ±0.02 km/s compared to the mean speed of −1.785 km/s for COSMOS2932. Similar corresponding values were obtained for the other four spatial objects targeted.

## 7. Conclusions

This article presented the CHEIA SST Radar, a new space tracking radar sensor developed and installed in Romania with the purpose of being included in the EU SST sensor network and of contributing to the joint database of space objects orbiting the Earth. The paper described the processes of design, simulation, and implementation of hardware and software building blocks that made up the radar system. It emphasized the particular case of using an already existing system of two large parabolic antennas that required an innovative design of retrofitting to function as the basis for a new quasi-monostatic radar using LFMCW probing signals. The preliminary design was validated by extensive simulations, and the initial operational testing carried out in December 2021 demonstrated the good performance of the radar in measuring the range and radial speed of LEO space objects. The radar tests proved the correct operation of the design even in highly detrimental conditions (low transmitted power and high phase noise). Because of this, the received data contained significant noise, but the final implementation of the radar will be able to precisely detect and track small targets according to the requirements of ROSA, the ESA, and the EU SST.

The comparison between the measured and filtered and the estimated ranges obtained during the initial tests for the COSMOS2932 satellite showed that the obtained results were very close to the theoretical ones, with the error being approximately ±0.6 km for range and ±0.02 km/s for Doppler speed. By increasing the *Tx* power and decreasing the phase noise of the PA, better accuracy of the radar measurements is expected.

The full configuration of the Cheia SST Radar will be completed during the beginning of 2022, with further works of installing a 5 kW SSPA, a tracking coupler block, and a monitoring system, as well as rack optimization. The commissioning of the system to initial operational status is ongoing and it is expected to be finalized in the first half of 2022. Several functional tests will be conducted in order to evaluate the full performance of the final configuration and to calibrate it according to EU SST procedures.

## Figures and Tables

**Table 1:** Main technical specifications of antennas in Cheia Communications Center.

Specification	Antenna Cheia 1	Antenna Cheia 2
Model	Intelsat Standard A Earth Station	
Manufacturer	Nippon Electric Co., Ltd. (NEC), Tokyo, Japan	
Diameter	32 m	
Year of installation	1976	1979
Satellite originally used	Intelsat IS—905 at 335.5° E	Intelsat IS—904 at 60.0° E
Total weight	309 ton	260 ton
Effective gain at feed	>63 dB	>63 dB
Noise temperature ԑ = 20°	37.0	36.5
Bandwidth	3.6–6.4 GHz	
Isolation between antenna sidelobes	>93 dB	
Polarization	Dual circular	
Polarization isolation	30 dB	
Azimuth scanning domain	−170° to +170° relative S	
Elevation scanning domain	0°–92°	
Tracking speed	0.3°/s	

**Table 2:** Measured ranges and radial speeds for spatial objects targeted during initial tests [33].

Object	Estimated *RCS* (m^2^)	Range Min (km)	Range Max (km)	Lowest Radial Speed (km/s)	Highest Radial Speed (km/s)
COSMOS2932	8.31	1496	2351	−1.43	−5.00
ENVISAT	19.49	818	2030	−0.45	−6.48
RADARSAT	3.62	849	2136	−1.42	−6.47
SS-18 R/B	9.18	966	2177	0	−6.09
DELTA 1 R/B	8.91	1108	2142	0	5.96
